# Psychosocial functioning mediates change in motor and cognitive function throughout neurorehabilitation for adults with acquired brain injury (ABI-RESTaRT)

**DOI:** 10.1007/s10072-023-06645-8

**Published:** 2023-02-13

**Authors:** Georgina Mann, Lakkhina Troeung, Krishneil A Singh, Curtis Reddell, Angelita Martini

**Affiliations:** 1 Brightwater Research Centre, Brightwater Care Group, Perth, Australia; 2 School of Psychological Science, University of Western Australia, Perth, Australia; 3 Oats Street Rehabilitation Centre, Brightwater Care Group, Perth, Australia; 4 State Head Injury Unit, Sir Charles Gairdner Hospital, Perth, Australia; 5 Transitional Accommodation Program, Brightwater Care Group, Perth, Australia

**Keywords:** Acquired brain injury, Neurorehabilitation, Post-acute, Community-based, Outcome measures, Mediation

## Abstract

**Objectives:**

This study aimed to evaluate the mediational role of change in psychosocial abilities, adjustment and participation on change in motor and cognitive function from admission to discharge from a staged community-based brain injury rehabilitation (SCBIR) service in Western Australia, 2011–2020.

**Methods:**

A retrospective cohort study of *n* = 324 adults with ABI enrolled in SCBIR using routinely collected rehabilitation outcome measures data. Motor and cognitive function were assessed with the UK Functional Independence and Assessment Measure and psychosocial function with the Mayo-Portland Adaptability Inventory-4. Six multilevel mediation regression analyses were conducted to determine whether change in psychosocial function (abilities, adjustment and participation) mediated change in motor and cognitive function from admission to discharge.

**Results:**

Participants demonstrated clinically significant improvements in both motor (+ 11.8, *p* < 0.001) and cognitive (+ 9.5, *p* < 0.001) functioning from admission to discharge. Statistically significant improvements in psychosocial abilities (− 4.8, *p* < 0.001), adjustment (− 2.9, *p* = 0.001) and participation (− 2.5, *p* < 0.001) were also seen but were not clinically significant. Mediation analyses showed that participation accounted for 81% of improvements in motor function at discharge and 71% of cognitive function improvements. Adjustment accounted for 26% and 32% of change in motor and cognitive function, respectively. Abilities accounted for 60% of change in cognitive function but did not significantly influence change in motor function. Changes in psychosocial participation fully mediated change in motor function during neurorehabilitation.

**Conclusions:**

Psychosocial function, particularly participation, is an important driver of motor and cognitive recovery throughout neurorehabilitation. Functional rehabilitation programs should target psychosocial improvement as an important mechanism of change.

**Supplementary Information:**

The online version contains supplementary material available at 10.1007/s10072-023-06645-8.

Acquired brain injury (ABI) is a leading cause of death and disability in Australia [1]. ABI can result from physical trauma (TBI) from an external source such as an assault, or non-traumatic causes (NTBI), such as stroke and hypoxia. ABI impacts functioning across multiple domains, leading to short- and long-term physical, cognitive, behavioural and psychosocial impairments impacting social engagement, self-care and quality of life [1–4].

Post-acute rehabilitation has been shown to significantly improve motor and cognitive functioning following ABI [5, 6]. However, despite the marked prevalence of psychosocial sequelae following ABI [7–10] and a considerable body of research demonstrating the negative impact of poor psychosocial functioning on rehabilitation engagement, recovery and quality of life [8, 9, 11, 12], the primary focus of most existing post-acute rehabilitation programs still remains motor and cognitive improvement [5, 6, 13], with psychosocial rehabilitation considered secondary.

Poor psychosocial function has a major impact on rehabilitation outcomes. Anxiety and depression after ABI has been linked with poorer participation [14, 15] and quality of life [16, 17]. Irritability and aggression have been associated with higher support needs [11]. Individuals with ABI and psychiatric comorbidity have also been shown to make poorer motor and cognitive improvement at rehabilitation discharge [8], despite remaining in services 6.5 months longer on average, compared to those without psychiatric comorbidity [8].

Psychosocial function is therefore intrinsically linked to functional recovery and should also be considered a primary target of neurorehabilitation [5]. However, while associations between poor psychosocial functioning, increased intervention needs and poorer rehabilitation outcomes have been established, research is yet to examine the specific role that psychosocial functioning plays in overall functional improvement post-ABI in terms of theoretical causal pathways and mechanisms of change [18]. This research is critical to allow researchers and clinicians to identify “active ingredients” of rehabilitation, establish causal pathways and design effective interventions. In particular, predictive mediation analysis is a statistical approach that can be used to identify mechanisms believed to underlie established relationships. Mediator variables lie along the pathway between a predictor and an outcome variable, explaining a proportion of the variance along that pathway and thus the nature of the relationship [18].

As part of the ABI-RESTaRT research program [19], this study aimed to investigate psychosocial functioning as a mediator of change in motor and cognitive function from admission to discharge in a retrospective cohort of adults with ABI undergoing staged community-based brain injury rehabilitation (SCBIR) [5] in Western Australia (WA), 2011–2020. We investigated two central questions: (1) Does change in psychosocial function mediate change in *motor* function from admission to discharge during post-acute rehabilitation? and (2) Does change in psychosocial function mediate change in *cognitive* function from admission to discharge during post-acute rehabilitation?

## Hypothesis

Two hypotheses were generated, each with three sub-hypotheses to allow testing:

- Hypothesis 1: Change in psychosocial function throughout rehabilitation mediates change in *motor* functional independence.
  - H1.1 Change in psychosocial *abilities* mediates motor change.
  - H1.2 Change in psychosocial *adjustment* mediates motor change.
  - H1.3 Change in psychosocial *participation* mediates motor change.
- Hypothesis 2: Change in psychosocial function throughout rehabilitation mediates change in *cognitive* functional independence.
  - H2.1 Change in psychosocial *abilities* mediates cognitive change.
  - H2.2 Change in psychosocial *adjustment* mediates cognitive change.
  - H2.3 Change in psychosocial *participation* mediates cognitive change.

## Method

### Ethics

Data were anonymised and collected as part of routine service provision, with clients providing prior written consent. Ethics approval was granted by the University of Western Australia Human Research Ethics Committee (HREC; RA/4/1/9232) and the Western Australia Department of Health HREC (RGS0000002894).

### Study design and cohort definition

ABI-RESTaRT is a retrospective whole-population cohort of adults aged 18–65 enrolled in post-acute community-based neurorehabilitation and support services (excluding respite) through the Brightwater Care Group in WA from 15 March 1991 to 31 December 2020 (*n* = 1011) [19]. Inclusion required a diagnosis of TBI, NTBI or eligible degenerative and non-degenerative neurological conditions defined by the Australian Rehabilitation Outcomes Centre (AROC) impairment codes [20].

This study evaluates functional outcomes for ABI-RESTaRT members enrolled in SCBIR between 1 January 2011 and 31 December 2020, when standardised outcome measures were introduced (*n* = 324). Of these, 211 clients (65%) had completed the Mayo-Portland Adaptability Inventory-4 (MPAI-4) and UK Functional Independence Measure and Functional Assessment Measure (FIM + FAM) at admission and discharge and were included for analysis. No significant differences in age, gender, time since injury or length of hospitalisation were identified between participants with and without outcome measure data.

### Setting

SCBIR is a multidisciplinary post-acute neurorehabilitation program [5] delivered at the 43-bed *Oats Street* rehabilitation centre in Perth [21]. SCBIR provides person-centred neurorehabilitation focusing on functional recovery. Clients are allocated to group homes of 4–5 residents based on functional capacity at admission. Houses range from 24-h support to independent living, supporting all stages of neurorehabilitation, from profound physical disability to higher level cognitive disability. Residents graduate through houses towards greater independence and reduced support as functional needs change (Figure S1). The program duration is 12–24 months.

### Data sources and extraction

Outcome measures were extracted through internal electronic medical records (EMRs). Clinical and demographic data were extracted from EMRs and probabilistically linked through the WA Data Linkage System [22] to a number of administrative data collections. Full details are elsewhere [19].

### Key measures

#### Demographic and clinical variables

Demographic and clinical data were extracted from EMRs and linked data including age, gender, relationship status, ABI diagnosis, injury date, cause of injury, acute hospitalisation dates, prior ABI, Australian Statistical Geography Standard (ASGS) Remoteness Area score [23] and Index of Relative Socioeconomic Disadvantage (IRSD) score [24]. Pre-admission residential postcodes were used to generate ASGS and IRSD scores, measuring geographical remoteness based on access to services from 1 (*Major Cities*) to 5 (*Very Remote*) and disadvantage based on education, employment and income across five quintiles from 1 (*most disadvantaged*) to 5 (*least disadvantaged*).

##### UK Functional Independence Measure and Functional Assessment Measure (FIM + FAM)

Functional independence was measured with the UK Functional Independence Measure and Functional Assessment Measure (FIM + FAM). FIM + FAM [25] is a 30-item measure of functional disability used to measure injury severity and rehabilitation support requirements. FIM + FAM measures functioning across two major domains: motor (16 items, e.g. self-care, transfers) and cognitive (14 items, e.g. communication, social cognition). Items are rated on a 7-point scale from 1 (*total assistance*) to 7 (*complete independence*) and summed to produce total scores, with higher scores representing greater independence. Clinically significant change was evaluated against published minimum clinically important difference (MCID) values [26]. MCID thresholds are 8.0 (motor), 7.0 (cognitive) and 15.0 (total FIM + FAM).

##### Mayo-Portland Adaptability Inventory-4 (MPAI-4)

Psychosocial functioning was measured using the Mayo-Portland Adaptability Inventory-4 (MPAI-4). MPAI-4 [27] is a 29-item measure of psychosocial disability, examining common sequelae of ABI across three major domains (see Table S1): abilities (12 items focused on functional disability across motor, cognitive and sensory domains, e.g. mobility, memory, vision), adjustment (12 items focused on emotional and behavioural sequelae of ABI, e.g. depression, aggression, impaired self-awareness) and participation (8 items focused on community participation and activities of daily living, e.g. initiation, transportation, social and recreational activities) on a scale ranging from 0 (*no limitation*) to 4 (*severe limitation*). Complete items in each domain can be seen in Table S1. Raw scores are calculated for the three domains and the total score, and then converted to standardised transformed scores (*T*-scores) based on normative data evaluating individuals undergoing community neurorehabilitation for ABI [27]. Higher *T*-scores indicate more severe psychosocial difficulties. The MCID is 5* T-*score points for total MPAI-4 [28]. The MPAI-4 is a widely used and well-validated instrument for the evaluation of psychosocial functioning in both TBI and stroke samples [27, 29] and displays adequate psychometric properties both for the use of the total score and the three major domains [27, 30]. Individual items within the scale are also considered valid for analysis [27].

### Statistical analysis

Data were analysed using STATA 16.1. Statistical significance was tested against alpha < 0.05 (uncorrected, two-tailed). Descriptive statistics are presented as mean (standard deviation), median (interquartile range) or count (percentage). Independent and paired-samples *t*-tests compared differences in continuous variables.

#### The mediational effect of psychosocial function on functional independence

To test the mediational effect of psychosocial function on functional independence over time (admission vs discharge), three-level multilevel mediation regression models were analysed. Multilevel models were used to control for potential bias in the longitudinal data, such as service delivery changes or random individual variation over time. Level 1 (lower level) data consisted of outcome measures during rehabilitation (FIM + FAM, MPAI-4) and was nested within level 2 data (participants), nested within level 3 data (service delivery period). An a priori power analysis was conducted, which indicated that the sample (*n* = 211) was sufficient to detect an anticipated medium effect (*f* = 0.25) at a power level of 0.8 with a design effect of 1.74 and 2 predictors, *n* = 74 [31].

#### Mediation pathways and criteria

**Fig. 1:**
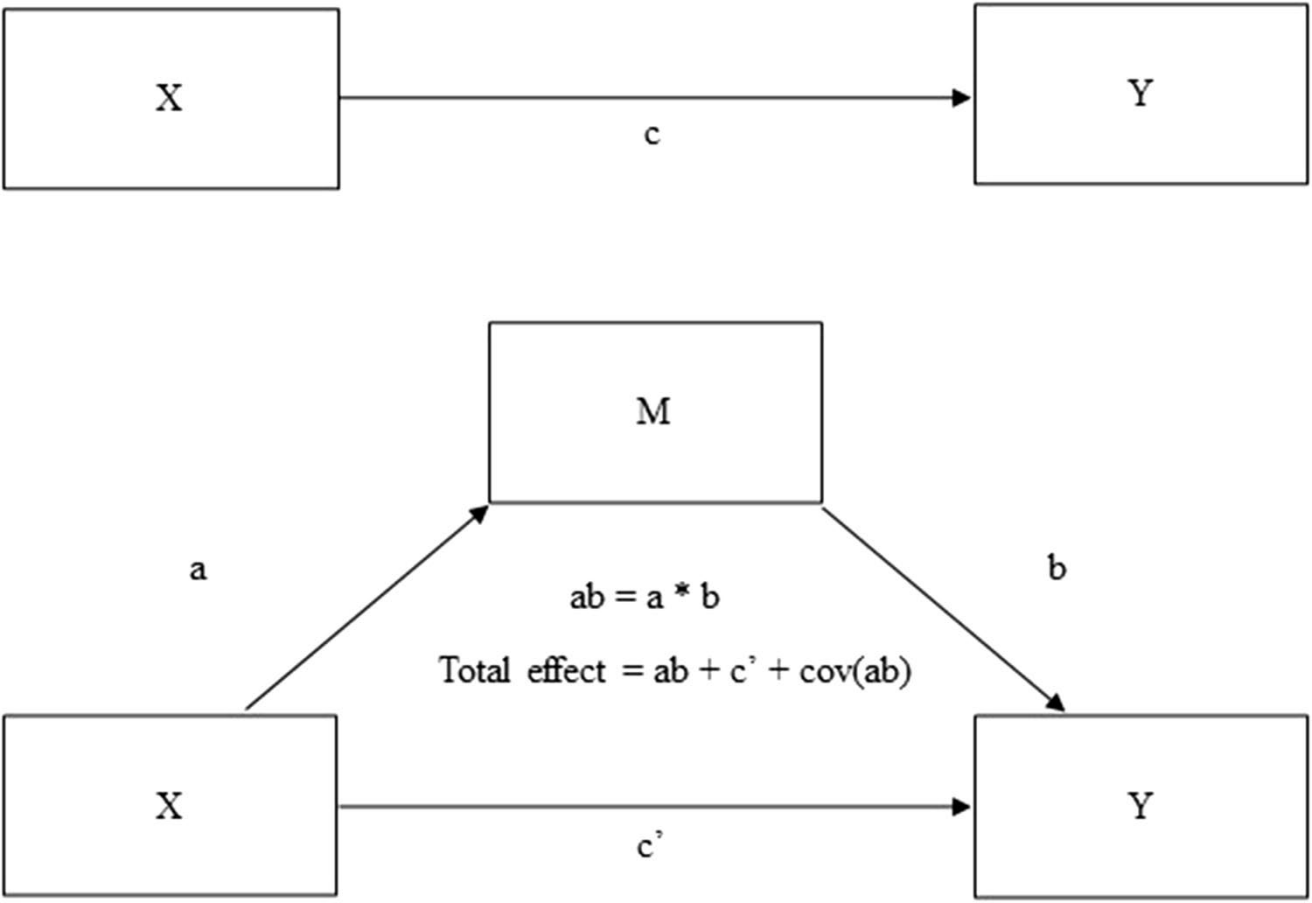
Generic mediation model indicating the relationships and paths between predictor (*X*), outcome (*Y*) and mediator (*M*)

Four statistical criteria must be present to establish mediation (Fig. 1) in accordance with Baron and Kenny’s guidelines [32, 33]:

1. The predictor (*X*) must be significantly related to the mediator (*M*) (path a).
2. The predictor (*X*) must be significantly related to the outcome (*Y*) (path c).
3. When the outcome, predictor and mediator are included in the model, the mediator must be significantly related to the outcome (path b).
4. The relationship between predictor and outcome with the mediator in the model (path c’) must be significantly reduced compared to when the outcome was regressed only on the predictor (path c).

#### Hypothesis testing

Six mediation models tested the hypothesised mediational role of each MPAI-4 domain (abilities, adjustment, participation) on change in FIM + FAM Motor (Hypothesis 1.1–1.3) and Cognitive (Hypothesis 2.1–2.3) scores throughout rehabilitation. Time (admission vs discharge) was the predictor variable (*X*). FIM + FAM score (motor and cognitive) was the outcome variable (*Y*). MPAI-4 scores (abilities, adjustment, participation) were the mediators (*M*).

The direct relationship between time (*X*) and FIM + FAM Motor and Cognitive was tested first to investigate whether functional independence changed from admission to discharge, and the direct path was significant (path c). Then, the mediator (MPAI-4; abilities, adjustment, participation) was added to test the indirect path (path ab) to determine whether the direct path significantly changed (path c’).

The strength of the mediation (and significance of the indirect pathway) was assessed using two methods. First, the Sobel test was used to measure the significance of the reduction in path c following the addition of the mediator (path c’), with an alpha ≤ 0.05 indicating a significant reduction [33]. Second, the percentage of the total effect accounted for by the indirect path was examined [32]. In a single level model, the total effect is calculated as ab + c’. However, for multilevel mediation, the total effect includes the covariance between paths a and b in the calculation of the total effect, e.g. Total effect = ab + c’ + covariance(ab) [32] (Fig. 1). Therefore, the percent mediation was calculated as 100 × total effect–c’/total effect, or 100 × (ab + c’ + covariance(ab))–c’/(ab + c’ + covariance(ab)).

#### Exploratory analyses

To evaluate the role of specific subdomains of psychosocial function on change in functional independence, exploratory analyses were conducted. In these analyses, individual items on the MPAI-4, evaluating specific domains of psychosocial function, were conducted using raw, non-transformed MPAI-4 scores. Each individual item was included in a mediation analysis, evaluating the effect of change in psychosocial function on change in motor and cognitive functional over time. The items used are detailed in Table S1. The analyses used were identical to those described above, but as the analyses were exploratory, all values should be interpreted with caution. No correction to the alpha level was used; therefore, results for the exploratory analyses were tested against an alpha < 0.05. The MPAI-4 was developed to allow the examination of the constructs measured as individual items [27]; therefore, this analysis was considered appropriate at the item level.

## Results

### Cohort characteristics

Characteristics of the SCBIR cohort are presented in Table 1. The majority were male (67.6%) with a mean age of 45.4 years (*SD* = 12.5 years). The most common diagnosis was NTBI (66.4%) followed by TBI (32.4%) and neurological conditions (1.2%). The median time from injury to admission was 8.7 months (IQR 5.3–17.6).

**Table 1:** Clinical and sociodemographic characteristics for ABI-RESTaRT clients in SCBIR (*n* = 324), stratified by diagnosis group

	Total, *n* = 324	TBI, *n* = 105	NTBI		Neurologic, *n* = 4
			Stroke, *n* = 137	Other, *n* = 78	
Sex,* n* (%)					
Male	219 (67.6)	85 (81.0)	95 (69.3)	37 (47.4)	2 (50.0)
Female	105 (32.4)	20 (19.1)	42 (30.7)	41 (52.6)	2 (50.0)
Age at admission, *M* (SD)	45.4 (12.5)	38.8 (13.4)	51.0 (9.3)	44.4 (12.2)	44.1 (6.6)
Age at injury, *M* (SD)	43.6 (13.4)	36.0 (13.9)	50.3 (9.5)	42.0 (13.1)	37.6 (5.1)
Aboriginal and/or Torres Strait Islander,* n* (%)	18 (5.6)	10 (9.5)	7 (5.1)	1 (1.3)	-
Resides in metropolitan area	257 (84.3)	82 (82.0)	109 (83.2)	62 (88.6)	4 (100)
IRSD disadvantage score Q4 or Q5	50 (15.4)	17 (16.2)	18 (13.1)	14 (18.0)	1 (25.0)
Partnered,* n* (%)	95 (29.3)	22 (21.0)	43 (31.4)	29 (37.2)	1 (25.0)
Time since injury, median [IQR] months	8.7 [5.3, 17.6]	12.4 [7.6, 20.5]	6.5 [4.6, 13.3]	8.4 [5.5, 12.7]	46.6 [9.2, 84.0]
Time since injury,* n* (%)					
Early: < 1 year	192 (60.6)	45 (42.9)	97 (71.3)	49 (66.2)	1 (50.0)
Middle: 1–2 years	59 (18.6)	31 (29.5)	21 (15.4)	7 (9.5)	-
Late: > 2 years	66 (20.8)	29 (27.6)	18 (13.2)	18 (24.3)	1 (50.0)
Injury location					
Bilateral	146 (45.1)	64 (61.0)	19 (13.9)	59 (75.6)	4 (100)
Left hemisphere	92 (28.4)	22 (21.0)	66 (48.2)	4 (5.1)	-
Right hemisphere	70 (21.6)	16 (15.2)	46 (33.6)	8 (10.3)	-
Unilateral–hemisphere unspecified	6 (1.9)	1 (1.0)	3 (2.2)	2 (2.6)	-
Unknown	10 (3.1)	2 (1.9)	3 (2.2)	5 (6.4)	-
Previous ABI, *n* (%)	37 (11.4)	8 (7.6)	24 (17.5)	5 (6.4)	0
Acute hospital admission length of stay, median [IQR] months	5.0 [2.8, 7.4]	5.3 [2.0, 8.6]	4.9 [2.9, 6.7]	5.0 [3.1, 7.5]	7.0 [4.7, 9.2]
Post-acute rehabilitation length of stay, median [IQR] months	19.6 [9.9, 33.3]	20.2 [9.7, 34.9]	22.8 [10.4, 30.7]	17.5 [10.6, 34.3]	15.1 [6.3, 33.2]

SCBIR staged community-based brain injury rehabilitation,* TBI* traumatic brain injury, *NTBI* non-traumatic brain injury, *SD* standard deviation, *IQR* interquartile rang﻿e

### Change in functional independence and psychosocial function at discharge

Admission, discharge and unadjusted change in FIM + FAM and MPAI-4 scores are presented in Table 2. Moderate limitations in cognitive and motor function were evidenced at admission. Participants were most impaired in abilities and adjustment domains (moderate-to-severe limitations), with mild-to-moderate limitations in participation. At discharge, participants showed clinically and statistically significant improvements in total FIM + FAM, motor and cognitive domains. Significant psychosocial improvements were also demonstrated in all four domains although improvements were not clinically significant.

**Table 2:** Unadjusted baseline FIM + FAM and MPAI-4 scores from admission to discharge (*n* = 211)

Outcome measure		Admission		Discharge		Change	Clinically significant
		*M*	SD	*M*	SD		
FIM + FAM	Motor	72.9	30.8	84.8	28.8	11.8**	Yes
	Cognitive	54.7	19.0	64.2	19.4	9.5**	Yes
	Total	127.7	45.4	149.0	45.4	21.3**	Yes
MPAI-4	Abilities	54.2	11.6	49.4	11.7	− 4.8**	-
	Adjustment	52.3	9.1	49.3	9.4	− 2.9*	-
	Participation	43.1	3.7	40.6	4.9	− 2.5**	-
	Total	49.2	8.5	45.4	9.2	− 3.8**	No

*FIM* + *FAM* Functional Independence Measure and Functional Assessment Measure, *MPAI-4* Mayo-Portland Adaptability Inventory-4

^*^Significant to *p* = 0.001

^**^Significant to *p* < 0.001

### Hypothesis 1: Mediators of change in motor function

Table 3 presents the results of the mediational analyses for change in motor function. Time significantly predicted motor function (path c), with participants showing significant motor gains throughout neurorehabilitation (*B* = 11.83, *p* < 0.001).

**Table 3:** Summary of level 1 regression for the mediation model for the cohort (*n* = 211) testing motor functional independence

Path	Predictor variable	Outcome variable	*B*	SE B	95% CI	Sobel *Z*	Path ab
C	Time	Motor function	11.83**	1.24	9.41, 14.25		
*Model 1: Change in motor function over time via psychosocial abilities*							
A	Time	Psychosocial abilities	− 4.79**	0.51	− 5.78, − 3.79	1.64	7.91
B	Psychosocial abilities	Motor function	− 1.65**	0.99	− 1.85, − 1.46		
C’	Time	Motor function	3.92*	1.25	1.46, 6.37		
*Model 2: Change in motor function over time via psychosocial adjustment*							
A	Time	Psychosocial adjustment	− 2.91**	0.54	− 3.97, − 1.86	4.51**	3.12**
B	Psychosocial adjustment	Motor function	− 1.07**	0.13	− 1.32, − 0.83		
C’	Time	Motor function	8.71**	1.24	6.28, 11.13		
*Model 3: Change in motor function over time via psychosocial participation*							
A	Time	Psychosocial participation	− 2.54**	0.23	− 2.99, − 2.08	8.56**	9.62**
B	Psychosocial participation	Motor function	− 3.79**	0.28	− 4.33, − 3.25		
C’	Time	Motor function	2.21	1.40	− 0.53, 4.95		

^*^Significant to *p* = 0.002

^**^Significant to *p* < 0.001

#### Hypothesis 1.1: Abilities

Psychosocial abilities significantly improved throughout rehabilitation (path a; *B* =  − 4.79, *p* < 0.001). Psychosocial abilities significantly predicted motor function (path b: *B* =  − 1.65, *p* < 0.001), indicating that those with better psychosocial abilities demonstrated greater motor function. With psychosocial abilities included, the coefficient for time predicting motor function reduced but remained significant (path c’; *B* = 3.92, *p* = 0.002). Sobel’s test was non-significant (path ab = 7.91), *Z* = 1.64, *p* = 0.10, indicating that change in psychosocial abilities did not significantly explain the relationship between time and motor function. Therefore, mediation was not present, and Hypothesis 1.1 was not supported.

#### Hypothesis 1.2: Adjustment

Psychosocial adjustment significantly improved throughout rehabilitation (path a; *B* =  − 2.91, *p* < 0.001). Psychosocial adjustment significantly predicted motor function (path b: *B* =  − 1.07, *p* < 0.001), indicating participants with better psychosocial adjustment demonstrated greater motor gains. When controlling for psychosocial adjustment, the coefficient for time predicting motor function was reduced but remained significant (path c’; *B* = 8.71, *p* < 0.001). Sobel’s test was significant (path ab = 3.12), *Z* = 4.51, *p* < 0.001, indicating that improvements in psychosocial adjustment *partially mediated* gains in motor function, with 26.5% of the relationship between time and motor function mediated by changes in psychosocial adjustment. Therefore, Hypothesis 1.2 was partially supported.

#### Hypothesis 1.3: Participation

Psychosocial participation significantly improved throughout rehabilitation (path a; *B* =  − 2.54, *p* < 0.001). Psychosocial participation significantly predicted motor function (path b: *B* =  − 3.79, *p* < 0.001), indicating those with better psychosocial participation demonstrated significant motor gains controlling for time. Controlling for psychosocial participation, time did not significantly predict motor function (path c’; *B* = 2.21, *p* = 0.114). Sobel’s test was significant (path ab = 9.63), *Z* = 8.56, *p* < 0.001, indicating that improvements in psychosocial participation *fully mediated* gains in motor function, with 81.3% of the relationship between time and motor function explained by changes in psychosocial participation. Therefore, Hypothesis 1.3 was supported.

### Hypothesis 2: Mediators of change in cognitive function

Table 4 shows the results of the mediational analyses for change in cognitive function. Time significantly predicted cognitive function (path c), with participants making significant cognitive gains during neurorehabilitation (*B* = 9.47, *p* < 0.001).

**Table 4:** Summary of the level 1 regression results for the mediation model for the cohort (*n* = 211) testing cognitive functional independence

Path	Predictor variable	Outcome variable	*B*	SE B	95% CI	Sobel *Z*	Path ab
C	Time	Cognitive function	9.47**	0.87	7.77, 11.16		
*Model 4: Change in cognitive function over time via psychosocial abilities*							
A	Time	Psychosocial abilities	− 4.79**	0.51	− 5.78, − 3.79	8.48**	5.63**
B	Psychosocial abilities	Cognitive function	− 1.18**	0.06	− 1.29, − 1.06		
C’	Time	Cognitive function	3.84**	0.87	2.14, 5.53		
*Model 5: Change in cognitive function over time via psychosocial adjustment*							
A	Time	Psychosocial adjustment	− 2.91**	0.54	− 3.97, − 1.85	4.98**	3.02**
B	Psychosocial adjustment	Cognitive function	− 1.04**	0.08	− 1.19, − 0.88		
C’	Time	Cognitive function	6.45**	0.81	4.87, 8.03		
*Model 6: Change in cognitive function over time via psychosocial participation*							
A	Time	Psychosocial participation	− 2.54**	0.23	− 2.99, − 2.08	9.00**	6.70**
B	Psychosocial participation	Cognitive function	− 2.64**	0.17	− 2.97, − 2.31		
C’	Time	Cognitive function	2.77*	0.91	0.98, 4.56		

^*^Significant to *p* = 0.002

^**^Significant to *p* < 0.001

#### Hypothesis 2.1: Abilities

Psychosocial abilities significantly improved throughout rehabilitation (path a; *B* =  − 4.79, *p* < 0.001). Psychosocial abilities significantly predicted cognitive function (path b; *B* =  − 1.18, *p* < 0.001), indicating that participants with greater psychosocial abilities demonstrated greater cognitive function. Controlling for psychosocial abilities, time remained a significant predictor of cognitive function (path c’; *B* = 3.84, *p* < 0.001). Sobel’s test was significant (path ab = 5.65), *Z* = 8.48, *p* < 0.001, indicating that improvements in psychosocial abilities *partially mediated* gains in cognitive function over time. Psychosocial abilities explained 59.4% of the relationship between time and cognitive function. Therefore, Hypothesis 2.1 was partially supported.

#### Hypothesis 2.2: Adjustment

Psychosocial adjustment significantly improved throughout rehabilitation (path a; *B* =  − 2.91, *p* < 0.001). Psychosocial adjustment also significantly predicted cognitive function (path b: *B* =  − 1.04, *p* < 0.001), indicating participants with better psychosocial adjustment demonstrated greater cognitive gains. Controlling for psychosocial adjustment, the coefficient for time predicting cognitive function was reduced but remained significant (path c’; *B* = 6.45, *p* < 0.001). Sobel’s test was significant (path ab = 3.02), *Z* = 4.98, *p* < 0.001, indicating that improvements in psychosocial adjustment *partially mediated* gains in cognitive function, with 31.9% of the relationship between time and cognitive function explained by psychosocial adjustment. Therefore, Hypothesis 2.2 was partially supported.

#### Hypothesis 2.3: Participation

Finally, psychosocial participation significantly improved throughout rehabilitation (path a: *B* =  − 2.54, *p* < 0.001). Psychosocial participation significantly predicted cognitive function (path b: *B* =  − 2.64, *p* < 0.001), indicating that participants with better psychosocial participation demonstrated greater cognitive function. Controlling for psychosocial adjustment, time significantly predicted cognitive function (path c’; *B* = 2.77, *p* < 0.001), with the path coefficient reduced but remaining significant. Sobel’s test was significant (path ab = 6.70), *Z* = 9.00, *p* < 0.001, indicating that improvements in psychosocial participation *partially mediated* gains in cognitive function, explaining 70.7% of the relationship between time and cognitive function. This partially supported Hypothesis 2.3.

### Exploratory analyses

Appendix 1 presents the results for the exploratory analyses. There was no evidence of full mediation for any MPAI-4 item, though a number of the individual items partially mediated the relationship between time and motor and cognitive functional independence. Results indicated that Motor function was most strongly partially mediated by mobility from the abilities domain (path ab = 6.89, Sobel *Z* = 6.94, *p* < 0.001), fatigue from the adjustment domain (path ab = 1.93, Sobel *Z* = 2.84, *p* = 0.005) and self-care from the participation domain (path ab = 7.89, Sobel *Z* = 6.27, *p* < 0.001). Cognitive function was most strongly partially mediated by novel problem-solving from the abilities domain (path ab = 3.68, Sobel *Z* = 5.51, *p* < 0.001), fatigue from the adjustment domain (path ab = 1.25, Sobel *Z* = 2.83, *p* = 0.005) and residence from the participation domain (path ab = 5.26, Sobel *Z* = 7.04, *p* < 0.001).

## Discussion

This study tested the mediational relationship between psychosocial functioning and motor and cognitive recovery in a retrospective cohort undergoing post-acute neurorehabilitation in WA. Overall, significant improvements in motor, cognitive and psychosocial function were seen at discharge. While improvements in psychosocial function were small, findings provide evidence that psychosocial function, particularly participation, plays a key role in functional improvement in neurorehabilitation.

Across all hypotheses, psychosocial participation demonstrated the greatest role in both motor and cognitive functional change throughout neurorehabilitation. Participation fully mediated motor functional change, explaining 81.3% of motor improvements, and partially mediated cognitive functional change, explaining 70.7% of cognitive improvements. Next, psychosocial adjustment partially mediated both motor functional change (explaining 26.3% of improvements) and cognitive functional change (explaining 31.9% of improvements). Finally, psychosocial abilities partially mediated cognitive change (explaining 59.4% of improvements), but did not mediate motor change. Our findings suggest that psychosocial function plays a greater role in motor and cognitive recovery than previously considered and that rehabilitation programs aiming to improve functional independence should target psychosocial function as an important mechanism of change. However, as no MPAI-4 domain explained 100% of change in motor or cognitive function, other mechanisms certainly influence functional improvement throughout neurorehabilitation.

Functional independence has long been a primary target of neurorehabilitation and commonly used to evaluate intervention effectiveness [5, 6, 13] and support requirements [5, 25]. However, psychosocial functioning is also crucial to consider in rehabilitation. Particularly, assessment of psychosocial functioning is critical to align rehabilitation interventions to client emotional status and readiness to ensure maximal participation based on impairment [5, 11]. Overall, our study supports prior research recommendations that psychosocial function and functional independence are both important targets in neurorehabilitation, as functional independence alone may not be sensitive to an individual’s changing needs [5]. The exact mechanism remains unclear; however, it is likely that poorer psychosocial function, including impaired social interaction and self-awareness, maladaptive emotional responses, withdrawal and low motivation [9, 16, 34–37], impacts readiness, engagement and tolerance of rehabilitation interventions, leading to poorer outcomes.

Our study highlights the particular importance of psychosocial participation in functional recovery after ABI. Individuals with initiation and motivation impairment, an important component of participation, report difficulties with goal-directed behaviours and apathy, leading to reduced engagement [36]. Consistent social and therapeutic participation enables individuals to learn and understand their own cognitive impairments, which may underlie improved cognitive functioning. Social contact, leisure and recreation activities are also important dimensions of participation that could drive cognitive and motor function. Engagement with recreational activities often declines following ABI [34], but maintenance and engagement with these activities is associated with better sense of self, mood, and quality of life [38]. Cognitive and physical skills are required to plan, engage and complete recreation activities, improving both cognitive and motor function. Participation in self-care (e.g., eating, grooming) is also frequently rehearsed throughout neurorehabilitation, involving repetition of cognitive and physical skills.

The relationship between psychosocial participation and functional gains in this cohort may directly reflect the SCBIR model, in which participation and integration into the local community is central [21]. This model focuses on creating an environment in which functional skills are learnt through real-world participation such as shopping, meal preparation, public transport training, social groups and engagement in community-based volunteering and employment. Additionally, the residential premises and group houses [21] provide opportunities for social participation and the practice of appropriate social skills.

Psychosocial adjustment partially mediated both cognitive and motor functional change, suggesting adjustment is an important driver of functional change in conjunction with other factors. Prior research has recognised psychosocial adjustment and emotional wellbeing as important contributors to change in neurorehabilitation [14, 39, 40]. Individuals with better emotional adjustment post-injury (e.g. low anxiety and no/reduced mood disturbance) demonstrated better clinical and medical outcomes following rehabilitation [15, 16, 35]. Specifically, emotional adjustment has been linked with rehabilitation readiness, a crucial factor in recovery. Impaired insight or denial of disability can impact realistic goal setting, adoption of compensatory strategies and motivation [40, 41], impacting rehabilitation outcomes. Adjustment to ABI can take years, may be non-linear and is sometimes never achieved. However, as adjustment improves, engagement may also improve, likely due to acceptance of changed functioning and the emergence of compensatory strategies. Interventions targeting different facets of adjustment, for example, improving mood, providing education about ABI, teaching emotion regulation and coping skills, are important for adjustment.

Psychosocial abilities partially mediated cognitive function but not motor function. The MPAI-4 psychosocial abilities domain primarily consists of items measuring executive functions (e.g. memory, attention) and therefore may have greater predictive value for cognitive outcomes than motor outcomes Overall, while participants made clinically meaningful functional gains from admission to discharge in our cohort, gains in psychosocial function did not reach clinical significance [28]. This indicates that psychosocial rehabilitation needs improvement to allow more meaningful gains to be achieved and, in turn, drive even greater improvements in functioning.

Finally, exploratory analyses identified mobility, fatigue and self-care as the individual psychosocial items which had the largest mediational effect on motor functional independence. This indicates that these areas are important rehabilitation targets to maximise motor functional improvement. Mobility is a core component of motor functioning as assessed by the FIM + FAM, with several items dedicated to the assessment of mobility, so it is unsurprising that change in mobility as assessed by the MPAI-4 is associated with change in mobility as assessed by the FIM + FAM. The self-care item evaluates an individual’s capacity to eat, dress and bathe, which is also assessed at length in the motor scale of the FIM + FAM across the first 6 items of the instrument. Therefore, it is possible that improvements in self-care are measured across both the MPAI-4 and the FIM + FAM. Fatigue is a common sequelae of ABI and has been associated with impaired executive functioning and quality of life [42]. These findings indicate that improvements in fatigue over time are associated with the capacity to make greater gains in both motor and cognitive function throughout neurorehabilitation, suggesting that improvements in fatigue may be associated with both alleviated cognitive dysfunction and reduced physical disability.

Novel problem-solving and residence were identified, along with fatigue, as the psychosocial items with the largest mediational effects on cognitive functional independence, indicating that these items are important targets for maximising cognitive functioning. Problem-solving is a complex activity involving numerous other cognitive processes, and so the improvement in problem-solving seen throughout neurorehabilitation is likely to be associated with more widespread cognitive gains, as seen in the present study. The residence item assesses an individual’s capacity to manage a household, including meal planning and medication management. This is a global cognitive function, requiring numerous skills such as attention and memory. Therefore, improvements in residence over time may be a driver of cognitive improvement through neurorehabilitation and an individual’s increasing independence. It is important to note that these findings were exploratory, and caution should be exercised in the interpretation of these results.

### Clinical implications

Psychosocial participation appears to drive both cognitive and motor functional independence in neurorehabilitation, supporting the use of rehabilitation models with built-in participation, like SCBIR. Community integration is critical to the development of psychosocial participation, with social activities involving engagement with both motor (e.g. using public transport) and cognitive (e.g. planning and remembering route) functioning. Findings also highlight the importance of targeting psychosocial adjustment in rehabilitation, as those with improved adjustment may be more receptive to interventions supporting functional gains. Changes in psychosocial adjustment occur slowly, evidenced by the small change seen in the sample, and should be targeted by rehabilitation clinicians as early as possible.

### Limitations

There are several limitations of the current study. Analyses did not adjust for clinical and demographic covariates that may impact neurorehabilitation outcomes, and a single mediator was tested in each model, preventing identification of other mechanisms of change. The inclusion of a single mediator allowed the hypotheses at test to be examined; however, this does not allow an understanding of the way these mediators influence one another throughout neurorehabilitation, and future research should aim to answer this question. Data was collected during routine clinical assessment, not under controlled settings. Therefore, while these findings are consistent with a causal explanation of the role of psychosocial function in functional change, other factors may be responsible. Finally, 65% of neurorehabilitation clients had complete data. While there were no significant differences in demographic or clinical characteristics between participants with and without data, there may be other differences between these two groups.

## Conclusions

Change in psychosocial participation fully mediates change in motor function and partially mediates change in cognitive function. Psychosocial adjustment and abilities explain a smaller proportion of variance in change in cognitive and motor function, suggesting neurorehabilitation should target psychosocial participation as a key mechanism of change. Future research should investigate the mechanisms by which psychosocial participation influences functional independence and develop programs to support psychosocial participation and improve outcomes from neurorehabilitation for ABI.

## Supplementary Information

Below is the link to the electronic supplementary material.

Supplementary file1 (DOCX 1.25 MB)

## Data Availability

The datasets presented in this article are not readily available due to organisational policies that restrict data sharing. Requests to access the datasets should be directed to the corresponding author.

## References

[1] Rushworth N (2008). Brain Injury Australia: submission to the Australian government’s national mental health and disability employment strategy.

[2] Fortune N, Wen X (1999). The definition, incidence and prevalence of acquired brain injury in Australia. AIHW cat. no. DIS 15.

[3] Fleminger S, Ponsford J (2005). Long term outcome after traumatic brain injury. BMJ.

[4] Seneviratne H (2022). The association between functional independence and quality of life for individuals with acquired brain injury undergoing community-based rehabilitation and disability support. NeuroRehabilitation.

[5] Jackson D (2017). Staged residential post-acute rehabilitation for adults following acquired brain injury: a comparison of functional gains rated on the UK Functional Assessment Measure (UK FIM+FAM) and the Mayo-Portland Adaptability Inventory (MPAI-4). Brain Inj.

[6] Williams E (2020). Time between acquired brain injury and admission to community-based rehabilitation: differences in cognitive and functional gains. Brain Inj.

[7] Jackson HM, Troeung L, Martini A (2020). Prevalence, patterns, and predictors of multimorbidity in adults with acquired brain injury at admission to staged community-based rehabilitation. Arch Rehabil Res Clin Transl.

[8] Troeung L (2022). Effects of comorbidity on post-acute outcomes in acquired brain injury: ABI-RESTaRT 1991–2020. Ann Phys Rehabil Med.

[9] Hammond FM (2019). Prevalence of medical and psychiatric comorbidities following traumatic brain injury. J Head Trauma Rehabil.

[10] Fisher LB (2016). Prevalence of suicidal behaviour following traumatic brain injury: longitudinal follow-up data from the NIDRR Traumatic Brain Injury Model Systems. Brain Inj.

[11] Sabaz M (2014). Prevalence, comorbidities, and correlates of challenging behavior among community-dwelling adults with severe traumatic brain injury: a multicenter study. J Head Trauma Rehabil.

[12] McCarthy ML (2006). Self-reported psychosocial health among adults with traumatic brain injury. Arch Phys Med Rehabil.

[13] Königs M (2018). Effects of timing and intensity of neurorehabilitation on functional outcome after traumatic brain injury: a systematic review and meta-analysis. Arch Phys Med Rehabil.

[14] Lewis FD, Horn GJ (2017). Depression following traumatic brain injury: impact on post-hospital residential rehabilitation outcomes. NeuroRehabilitation.

[15] Horn G, Lewis FD, Russell R, Kemp D (2017) Anxiety following traumatic brain injury: impact on post hospital rehabilitation outcomes. Phys Med Rehabil Res 2:1–6. 10.15761/PMRR.1000149

[16] Bombardier CH (2010). Rates of major depressive disorder and clinical outcomes following traumatic brain injury. JAMA.

[17] Rafsten L, Danielsson A, Sunnerhagen KS (2018). Anxiety after stroke: a systematic review and meta-analysis. J Rehabil Med.

[18] Field-Fote E (2019). Mediators and moderators, confounders and covariates: exploring the variables that illuminate or obscure the “active ingredients” in neurorehabilitation. J Neurol Phys Ther.

[19] Mann G et al (2021) Cohort profile: the Acquired Brain Injury Community REhabilitation and Support Services OuTcomes CohoRT (ABI-RESTaRT), Western Australia, 1991–2020. BMJ Open 11(9):e052728 10.1136/bmjopen-2021-052728 PMC8413932 34475189

[20] Australian Rehabilitation Outcomes Centre (2013). AROC impairment coding guidelines.

[21] Brightwater Care Group (2018) Rehabilitation for life: the oats street model for acquired brain injury rehabilitation. Available online at: https://brightwatergroup.com/media/1446/brightwater-oats-street-brochure.pdf. Accessed 4 Sep 2020

[22] Department of Health Government of Western Australia (2020) Data Linkage Western Australia. Available online at: https://www.datalinkage-wa.org.au/. Accessed 4 Aug 2022

[23] Australian Bureau of Statistics (2018) The Australian Statistical Geography Standard (ASGS) Remoteness Structure. Available online at: https://www.abs.gov.au/websitedbs/D3310114.nsf/home/remoteness+structure. Accessed 10 Jan 2021

[24] Australian Bureau of Statistics (2018) Socio-economic indexes for areas. Available online at: https://www.abs.gov.au/websitedbs/censushome.nsf/home/seifa. Accessed 10 Jan 2021

[25] Turner-Stokes L (1999). The UK FIM+FAM: development and evaluation. Clin Rehabil.

[26] Wilson FC, Wheatley-Smith L, Downes C (2009). Analysis of intensive outpatient neuro-rehabilitation outcomes using FIM+ FAM UK. NeuroRehabilitation.

[27] Malec JF, Lezak MD (2003) Manual for the Mayo-Portland Adaptability Inventory (MPAI-4) for adults, children and adolescents. Indianapolis, Indiana

[28] Malec JF, Kean J, Monahan PO (2017). The minimal clinically important difference for the Mayo-Portland Adaptability Inventory (MPAI-4). J Head Trauma Rehabil.

[29] Malec JF (2012). Mayo-Portland Adaptability Inventory: comparing psychometrics in cerebrovascular accident to traumatic brain injury. Arch Phys Med Rehabil.

[30] Kean J (2011). Rasch measurement analysis of the Mayo-Portland Adaptability Inventory (MPAI-4) in a community-based rehabilitation sample. J Neurotrauma.

[31] Snijders TA (2005). Power and sample size in multilevel modeling. Encycl Stat Behav Sci.

[32] Kenny DA, Korchmaros JD, Bolger N (2003). Lower level mediation in multilevel models. Psychol Methods.

[33] Baron RM, Kenny DA (1986). The moderator–mediator variable distinction in social psychological research: conceptual, strategic, and statistical considerations. J Pers Soc Psychol.

[34] Wise EK (2010). Impact of traumatic brain injury on participation in leisure activities. Arch Phys Med Rehabil.

[35] Brandel MG (2017). The association between psychiatric comorbidities and outcomes for inpatients with traumatic brain injury. J Neurotrauma.

[36] Palmisano S, Fasotti L, Bertens D (2020). Neurobehavioral initiation and motivation problems after acquired brain injury. Front Neurol.

[37] Hurst FG (2020). Domain-specific deficits in self-awareness and relationship to psychosocial outcomes after severe traumatic brain injury. Disabil Rehabil.

[38] Tate R, Wakim D, Genders M (2014). A systematic review of the efficacy of community-based, leisure/social activity programmes for people with traumatic brain injury. Brain Impair.

[39] Cicerone KD (2008). A randomized controlled trial of holistic neuropsychologic rehabilitation after traumatic brain injury. Arch Phys Med Rehabil.

[40] Prigatano GP, Sherer M (2020). Impaired self-awareness and denial during the postacute phases after moderate to severe traumatic brain injury. Front Psychol.

[41] Katz N (2002). Unawareness and/or denial of disability: implications for occupational therapy intervention. Can J Occup Ther.

[42] Åkerlund E, Sunnerhagen KS, Persson HC (2021). Fatigue after acquired brain injury impacts health-related quality of life: an exploratory cohort study. Sci Rep.

