# Supplementary material for: Psychosocial functioning mediates change in motor and cognitive function throughout neurorehabilitation for adults with acquired brain injury (ABI-RESTaRT)
Source: Neurol Sci. 2023 Feb 13;44(7):2401–11. doi: 10.1007/s10072-023-06645-8 (PMC10257595; doi:10.1007/s10072-023-06645-8)
Supplement: Supplementary file 1 — Supplementary file1 (DOCX 1.25 MB) [file 10072_2023_6645_MOESM1_ESM.docx]

#
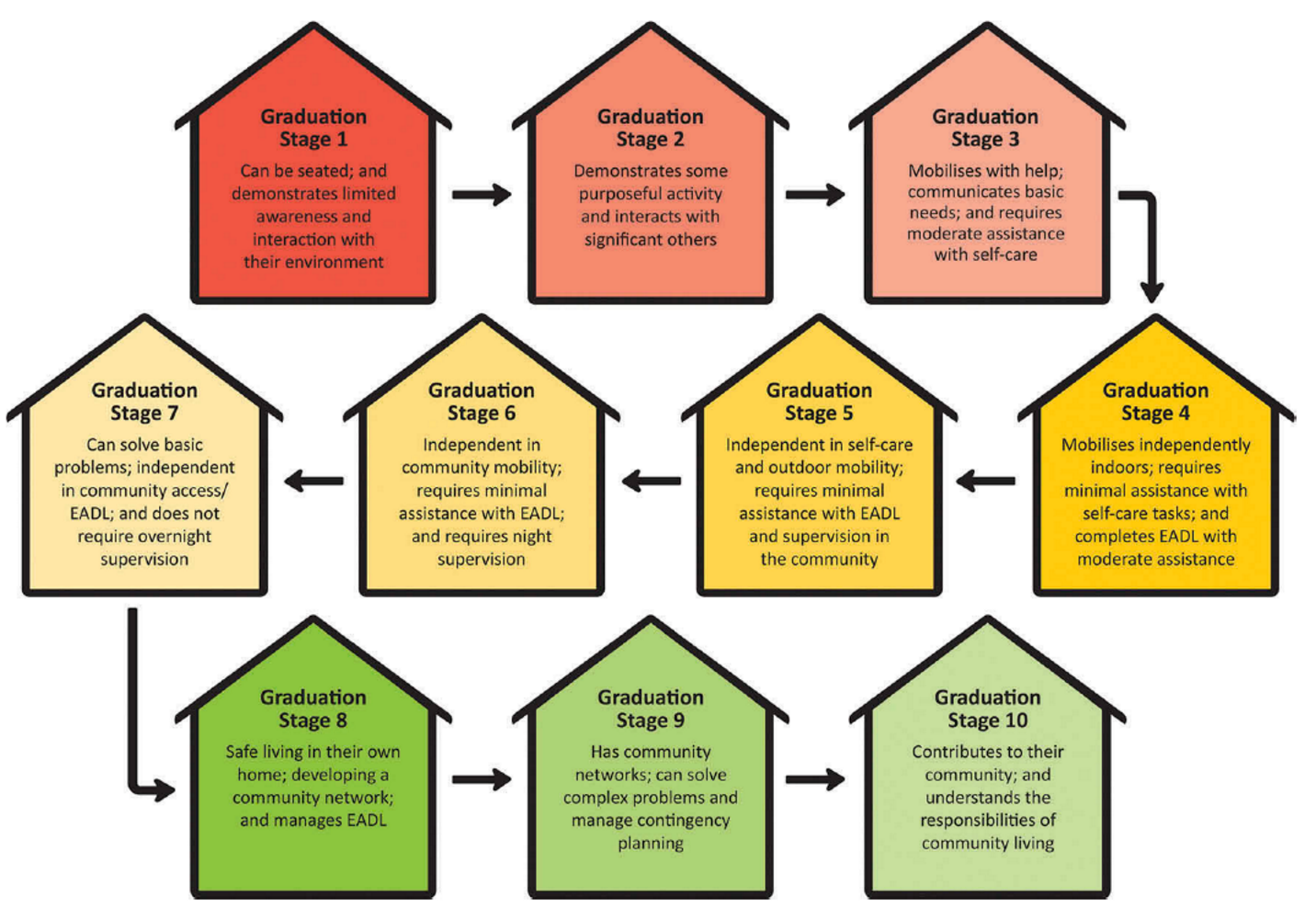
Supplementary Materials

**Figure S 1. Staged Community-Based Brain Injury Rehabilitation (SCBIR) Graduation Stages**

## Table S1.

Summary of MPAI-4 items included in each of the three domains: Abilities, Adjustment and Participation.

| Item | Item Label | Description |
| --- | --- | --- |
| Part A: Abilities | | |
| 1 | Mobility | Difficulties with walking or balance |
| 2 | Use of hands | Weakness or impaired coordination |
| 3 | Vision | Vision impairments |
| 4 | Audition* | Hearing impairments |
| 5 | Dizziness | Dizziness or light-headedness |
| 6 | Motor speech | Speech impairment, stuttering |
| 7A | Verbal communication | Difficulties with expressive or receptive language |
| 7B | Nonverbal communication | Unusual gesture or facial expression, missing non-verbal cues |
| 8 | Attention/Concentration | Problems with distraction, attentional shifting or working memory |
| 9 | Memory | Memory difficulties |
| 10 | Fund of information | Difficulties remembering previously learned information or information about self and family |
| 11 | Novel problem-solving | Difficulties solving problems |
| 12 | Visuospatial Abilities | Difficulties with visual awareness, drawing and route-finding |
| Part B: Adjustment | | |
| 13 | Anxiety | Experiencing anxiety symptoms |
| 14 | Depression | Experiencing depressive symptoms |
| 15 | Irritability, anger, aggression | Verbal or physical expressions of anger |
| 16 | Pain and headache* | Expressions of pain or activity limitation |
| 17 | Fatigue | Low energy, easily fatigued |
| 18 | Sensitivity to mild symptoms | Concern or worry over symptoms attributed to brain injury |
| 19 | Inappropriate social interaction | Inappropriate behaviour |
| 20 | Impaired self-awareness | Lack of awareness of limitations |
| 21 | Family/significant relationships | Stress relating to family functioning and household tasks |
| Part C: Participation | | |
| 22 | Initiation^ | Difficulties with initiation |
| 23 | Social contact with friends, work associates, and other people who are not family, significant others, or professionals^ | Difficulties with social interaction |
| 24 | Leisure and recreational activities^ | Difficulties with activities and recreation |
| 25 | Self-care | Completion of eating, dressing, bathing activities |
| 26 | Residence | Independent living responsibilities, like meal preparation, medication management |
| 27* | Transportation | Capacity to drive or use public transport |
| 28A* | Paid employment | Hours of paid employment and need for support |
| 28B* | Other employment | Constructive, role-appropriate activity other than paid work, eg. Study. |
| 29 | Managing money and finances | Managing personal finances, managing money. |

*Indicates items that require rescoring as specified in the MPAI-4 manual before raw scores are summed.

^These items are included in both Adjustment and Participation indices

Note: MPAI-4 includes a Part D: pre-existing and associated conditions, which does not contribute to the calculation of the total score, and as such was not included in the present study.

# Appendix 1

## Exploratory Analysis

To determine the role of individual domains of psychosocial function on change in cognitive and motor functional independence over time, exploratory analyses were conducted. These analyses examined the mediational effect of each individual MPAI-4 item on change in motor and cognitive function. Analyses of individual items of the MPAI-4 are presented within tables, grouped within their original domains (Abilities, Adjustment, Participation) as presented in Table S1. Analyses can be seen for each domain of the MPAI-4 and both Motor (Tables S2-S4) and Cognitive (Tables S5-S7) functional independence.

Where the first three of the four statistical criteria required to establish mediation [32, 33] were not present (Figure 1), neither Sobel tests nor the indirect path (path ab) were calculated in the models. The first criterion, that the predictor (X) must be significantly related to the mediator (M) (path a), was violated for a number of the mediation models: Time did not significantly predict change in Anxiety, Depression, Sensitivity to Mild Symptoms, Inappropriate Social Interaction, Family and Significant Relationships or Paid and Other Employment. As such, mediation was not tested. The second criterion, that the predictor (X) must be significantly related to the outcome (Y) (path c) was established, as both Motor function and Cognitive function were significantly related to Time. The third criterion, that when the outcome, predictor and mediator are included in the model, the mediator must be significantly related to the outcome (path b), was also violated for one of the models: Cognitive function did not predict Dizziness. As such, mediation was not evaluated for Dizziness. The fourth and final criterion, that the relationship between predictor and outcome with the mediator in the model (path c’) must be significantly reduced compared to when the outcome was regressed only on the predictor (path c) was not supported. This indicates that full mediation was not present in any of the exploratory analyses.

## Motor Function

Time significantly predicted Motor function (path c), with participants showing significant gains throughout rehabilitation (*B*=11.83, *p*<0.001).

### Abilities

Table S2 presents the results of the mediational analyses for change in Motor function in the items of the Abilities domain. Throughout, it appeared that change in all individual items of the Abilities domain partially mediated change in Motor function over Time, with the exception of Audition, for which there was no evidence of mediation. Of the items in the Abilities domain, Mobility explained the greatest percentage of the relationship between Time and Motor function, with 58.27% of the relationship explained by Mobility.

### Adjustment

Table S3 presents the results of the mediational analyses for change in Motor function in the items of the Adjustment domain. At the item level, mediation was not evaluated for Anxiety, Depression, Sensitivity to Mild Symptoms, Inappropriate Social Interaction or Family/Significant Relationships, as they violated the first criteria of mediation. Neither Irritability, Anger, and Aggression nor Impaired Self-Awareness provided evidence of mediation of the relationship between Time and Motor function. Pain and Headache, and Fatigue both provided evidence of *partial mediation* of the relationship between Time and Motor Function. Fatigue explained the greatest percentage of mediation, with 16.32% of the relationship between Time and Motor function explained by Fatigue.

### Participation

Table S4 presents the results of the mediational analyses for change in Motor functions for the individual items of the Participation domain. At the item level, mediation was not evaluated for Paid or Other Employment, as this item violated the first criterion of mediation. Initiation, Social Contact, Leisure and Recreation, Self-Care, Residence, Transportation, and Managing Money each demonstrated evidence of *partial mediation* of the relationship between Time and Motor function. Self-Care explained the greatest percentage of the Participation domain, with 66.68% of the relationship between Time and Motor function explained by Self-Care.

## Cognitive Function

Time significantly predicted change in Cognitive function (path c), with participants showing significant gains in cognitive function throughout rehabilitation (*B=*9.47, *p*<0.001).

### Abilities

Table S5 presents the results of the mediational analyses for change in Cognitive function in the items of the Abilities domain. At the item level, mediation analysis was not evaluated for Dizziness, as this violated the third criteria of mediation. Neither change in Vision nor Audition demonstrated mediation of the relationship between change in Cognitive function over Time. Mobility, Use of hands, Motor Speech, Verbal and Nonverbal Communication, Attention, Memory, Fund of Information, Novel Problem Solving and Visuospatial Abilities all demonstrated evidence of *partial mediation.* Novel problem-solving explained the greatest percentage of mediation, with 38.83% of the relationship between Time and Cognitive function explained by Novel problem-solving.

### Adjustment

The mediational analysis for change in Cognitive function and items of the Adjustment domain are presented in Table S6. At the item level, mediation analyses were not conducted for Anxiety, Depression, Sensitivity to Mild Symptoms, Inappropriate Social Interaction or Family/Significant Relationships, as they violated the first criteria of mediation as described above. There was no evidence of Impaired Self-Awareness mediating the relationship between Time and Cognitive function. Irritability, Anger and Aggression, Pain and Headache and Fatigue demonstrated evidence of a partial mediation of the relationship between Time and Cognitive function. There was no evidence of full mediation across any individual items of the Adjustment domain. Of the items in the Adjustment domain, Fatigue also explained the greatest percentage of the relationship between Time and Cognitive function, with 13.25% of the relationship explained by Fatigue.

### Participation

Table S7 presents the mediation analyses for change in Cognitive function and the items of the Participation domain. As described above, mediation was not evaluated for Paid or Other Employment, as this item violated the first criterion of mediation. There was evidence that Initiation, Social Contact, Leisure and Recreation, Self-Care, Residence, Transportation and Managing Money demonstrated *partial mediation* of the relationship between Time and Cognitive function. Of the Participation domain, Residence explained the greatest percentage of mediation, with 55.56% of the relationship between Time and Cognitive function explained by Residence.

**Table S2. Summary of the Level 1 Regression results testing the Mediational Effects of MPAI-4 Abilities Domain Items on Change in FIM+FAM Motor Score (n=211)**

| Path | Predictor Variable | Outcome Variable | B | SE B | 95% CI | Sobel Z | Path ab |
| --- | --- | --- | --- | --- | --- | --- | --- |
| C | Time | Motor function | 11.83** | 1.24 | 9.41, 14.25 |  |  |
| *Item 1: Mobility* | | | | | | |  |
| A | Time | Mobility | -0.44** | 0.06 | -0.56, -0.31 | 6.94** | 6.89** |
| B | Mobility | Motor function | -15.80** | 0.74 | -17.25, -14.35 |  |  |
| C’ | Time | Motor function | 4.94** | 1.09 | 2.81, 7.07 |  |  |
| *Item 2: Use of Hands* | | | | | | |  |
| A | Time | Use of hands | -0.40** | 0.06 | -0.52, -0.29 | 6.19** | 5.96** |
| B | Use of hands | Motor function | -14.80** | 0.89 | -16.53, -13.06 |  |  |
| C’ | Time | Motor function | 5.87** | 1.26 | 3.39, 8.35 |  |  |
| *Item 3: Vision* | | | | | | |  |
| A | Time | Vision | -.31** | 0.06 | -4.33, -0.18 | 3.92** | 1.93** |
| B | Vision | Motor function | -6.25** | 1.04 | -8.30, -4.21 |  |  |
| C’ | Time | Motor function | 9.90** | 1.28 | 7.40, 12.41 |  |  |
| *Item 4: Audition* | | | | | | | |
| A | Time | Audition | -0.13* | 0.06 | -0.24, -0.02 | 1.89 | 0.64* |
| B | Audition | Motor function | -5.02** | 1.30 | -7.56, -2.48 |  |  |
| C’ | Time | Motor function | 11.19** | 1.24 | 8.77,13.61 |  |  |
| *Item 5: Dizziness* | | | | | | | |
| A | Time | Dizziness | -0.26** | 0.06 | -0.38, -0.14 | 2.81* | 1.15* |
| B | Dizziness | Motor function | -4.42** | 1.20 | -6.76, -2.08 |  |  |
| C’ | Time | Motor function | 10.68** | 1.26 | 8.21, 13.15 |  |  |
| *Item 6: Motor Speech* | | | | | | | |
| A | Time | Motor Speech | -0.22** | 0.05 | -0.32, -0.12 | 4.11** | 2.27** |
| B | Motor Speech | Motor function | -10.39** | 0.90 | -12.14, -8.64 |  |  |
| C’ | Time | Motor function | 9.56** | 1.14 | 7.34, 11.79 |  |  |
| *Item 7: Verbal or Nonverbal Communication* | | | | | | | |
| A | Time | Verbal or Nonverbal Communication | -.33** | 0.06 | -0.44, -0.22 | 4.54** | 2.85** |
| B | Verbal or Nonverbal Communication | Motor function | -8.59** | 1.07 | -10.68, -6.50 |  |  |
| C’ | Time | Motor function | 8.98** | 1.22 | 6.59, 11.37 |  |  |
| *Item 8: Attention* | | | | | | | |
| A | Time | Attention | -0.29** | 0.07 | -0.44, -0.15 | 3.46** | 1.90* |
| B | Attention | Motor function | -6.47** | 1.03 | -8.50, -4.45 |  |  |
| C’ | Time | Motor function | 9.93** | 1.27 | 7.45, 12.41 |  |  |
| *Item 9: Memory* | | | | | | | |
| A | Time | Memory | -0.37** | 0.06 | -0.49, -0.26 | 3.88** | 2.15** |
| B | Memory | Motor function | -5.75** | 1.15 | -8.00, -3.50 |  |  |
| C’ | Time | Motor function | 9.68** | 1.30 | 7.13,12.22 |  |  |
| *Item 10: Fund of Information* | | | | | | | |
| A | Time | Fund of Information | -0.45** | 0.08 | -0.60, -0.30 | 4.58** | 3.22** |
| B | Fund of Information | Motor function | -7.16** | 0.91 | -8.93, -5.38 |  |  |
| C’ | Time | Motor function | 8.61** | 1.28 | 6.09,11.12 |  |  |
| *Item 11: Novel Problem Solving* | | | | | | | |
| A | Time | Novel problem-solving | -0.37** | 0.06 | -0.48, -0.26 | 4.54** | 3.10** |
| B | Novel problem-solving | Motor function | -8.39** | 1.25 | -10.84, -5.95 |  |  |
| C’ | Time | Motor function | 8.73** | 1.32 | 6.13, 11.32 |  |  |
| *Item 12: Visuospatial Abilities* | | | | | | | |
| A | Time | Visuospatial Abilities | -0.35** | 0.07 | -0.49, -0.21 | 4.44** | 3.18** |
| B | Visuospatial Abilities | Motor function | -9.19** | 0.95 | -11.05, -7.33 |  |  |
| C’ | Time | Motor function | 8.65** | 1.29 | 6.13, 11.17 |  |  |

*Significant to *p<*0.05; **Significant to *p<*0.001

**Table S3. Summary of the Level 1 Regression results testing the Mediational Effects of MPAI-4 Adjustment Domain Items on Change in FIM+FAM Motor Score (n=211)**

| Path | Predictor Variable | Outcome Variable | B | SE B | 95% CI | Sobel Z | Path ab |
| --- | --- | --- | --- | --- | --- | --- | --- |
| C | Time | Motor function | 11.83** | 1.24 | 9.41, 14.25 |  |  |
| *Item 13: Anxiety* | | | | | | |  |
| A | Time | Anxiety | -0.15 | 0.78 | -0.30, <0.001 | - | - |
| B | Anxiety | Motor function | -3.10* | 0.96 | -4.98, -1.22 |  |  |
| C’ | Time | Motor function | 11.36** | 1.24 | 8.92, 13.79 |  |  |
| *Item 14: Depression* | | | | | | |  |
| A | Time | Depression | -0.05 | 0.08 | -0.20, 0.10 | - | - |
| B | Depression | Motor function | -2.92* | 0.98 | -4.83, -1.00 |  |  |
| C’ | Time | Motor function | 11.69** | 1.23 | 9.28, 14.10 |  |  |
| *Item 15: Irritability, Anger, Aggression* | | | | | | |  |
| A | Time | Irritability, Anger, Aggression | 0.18* | 0.08 | 0.03, 0.33 | 1.67 | -0.44 |
| B | Irritability, Anger, Aggression | Motor function | -2.42* | 0.97 | -4.31, -0.52 |  |  |
| C’ | Time | Motor function | 12.27** | 1.25 | 9.81, 14.72 |  |  |
| *Item 16: Pain and Headache* | | | | | | | |
| A | Time | Pain and Headache | -0.22* | 0.07 | -0.36, -0.08 | 2.77* | 1.30* |
| B | Pain and Headache | Motor function | -5.84** | 1.00 | -7.80, -3.88 |  |  |
| C’ | Time | Motor function | 10.53** | 1.23 | 8.13, 12.93 |  |  |
| *Item 17: Fatigue* | | | | | | | |
| A | Time | Fatigue | -0.21* | 0.07 | -0.35, -0.08 | 2.84* | 1.93* |
| B | Fatigue | Motor function | -9.03** | 1.03 | -11.05, -7.01 |  |  |
| C’ | Time | Motor function | 9.91** | 1.22 | 7.51, 12.30 |  |  |
| *Item 18: Sensitivity to Mild Symptoms* | | | | | | | |
| A | Time | Sensitivity to Mild Symptoms | -0.07 | 0.08 | -0.23, 0.10 | - | - |
| B | Sensitivity to Mild Symptoms | Motor function | -2.85* | 0.89 | -4.59, -1.12 |  |  |
| C’ | Time | Motor function | 11.64** | 1.22 | 9.25, 14.03 |  |  |
| *Item 19: Inappropriate Social Interaction* | | | | | | | |
| A | Time | Inappropriate Social Interaction | 0.10 | 0.08 | -0.07, 0.26 | - | - |
| B | Inappropriate Social Interaction | Motor function | -2.39* | 0.90 | -4.16, -0.62 |  |  |
| C’ | Time | Motor function | 12.06** | 1.24 | 9.63, 14.48 |  |  |
| *Item 20: Impaired Self-Awareness* | | | | | | | |
| A | Time | Impaired Self-Awareness | 0.16* | 0.08 | -0.32, <-0.001 | 1.46 | 0.31 |
| B | Impaired Self-Awareness | Motor function | -1.92* | 0.90 | -3.69, -0.15 |  |  |
| C’ | Time | Motor function | 11.52** | 1.24 | 9.10, 13.95 |  |  |
| *Item 21: Family/Significant Relationships* | | | | | | | |
| A | Time | Family/Significant Relationships | -0.17 | 0.09 | -0.35, 0.009 | - | - |
| B | Family/Significant Relationships | Motor function | -2.54* | 0.82 | -4.15, -0.94 |  |  |
| C’ | Time | Motor function | 11.40** | 1.23 | 8.99, 13.80 |  |  |

*Significant to *p<*0.05; **Significant to *p<*0.001

**Table S4. Summary of the Level 1 Regression results testing the Mediational Effects of MPAI-4 Participation Domain Items on Change in FIM+FAM Motor Score (n=211)**

| Path | Predictor Variable | Outcome Variable | B | SE B | 95% CI | Sobel Z | Path ab |
| --- | --- | --- | --- | --- | --- | --- | --- |
| C | Time | Motor function | 11.83** | 1.24 | 9.41, 14.25 |  |  |
| *Item 22: Initiation* | | | | | | |  |
| A | Time | Initiation | -0.45** | 0.08 | -0.61, -0.29 | 4.82** | 3.54** |
| B | Initiation | Motor function | -7.95** | 0.85 | -9.61, -6.29 |  |  |
| C’ | Time | Motor function | 8.29** | 1.23 | 5.88, 10.70 |  |  |
| *Item 23: Social Contact* | | | | | | |  |
| A | Time | Social Contact | -0.56** | 0.09 | -0.72, -0.39 | 4.54** | 3.23** |
| B | Social Contact | Motor function | -5.83** | 0.88 | -7.56, -4.10 |  |  |
| C’ | Time | Motor function | 8.60** | 1.31 | 6.02, 11.17 |  |  |
| *Item 24: Leisure and Recreation* | | | | | | |  |
| A | Time | Leisure and Recreation | -0.79** | 0.08 | -0.94, -0.64 | 5.87** | 5.86** |
| B | Leisure and Recreation | Motor function | -7.45** | 1.02 | -9.45, -5.45 |  |  |
| C’ | Time | Motor function | 5.97** | 1.46 | 3.11, 8.82 |  |  |
| *Item 25: Self-Care* | | | | | | | |
| A | Time | Self-Care | -0.53** | 0.08 | -0.68, -0.38 | 6.27** | 7.89** |
| B | Self-Care | Motor function | -14.86** | 0.77 | -16.36, -13.35 |  |  |
| C’ | Time | Motor function | 3.94* | 1.25 | 1.50, 6.38 |  |  |
| *Item 26: Residence* | | | | | | | |
| A | Time | Residence | -0.66** | 0.07 | -0.80, -0.53 | 6.72** | 7.31** |
| B | Residence | Motor function | -11.02** | 1.15 | -13.28, -8.76 |  |  |
| C’ | Time | Motor function | 4.52* | 1.46 | 1.65, 7.39 |  |  |
| *Item 27: Transportation* | | | | | | | |
| A | Time | Transportation | -0.60** | 0.06 | -0.73, -0.48 | 5.33** | 4.51** |
| B | Transportation | Motor function | -7.49** | 1.19 | -9.82, -5.15 |  |  |
| C’ | Time | Motor function | 7.32** | 1.45 | 4.47, 10.18 |  |  |
| *Item 28: Paid or Other Employment* | | | | | | | |
| A | Time | Paid or Other Employment | -0.06 | 0.04 | -0.14, 0.02 | - | - |
| B | Paid or Other Employment | Motor function | -5.14* | 1.67 | -8.41, -1.87 |  |  |
| C’ | Time | Motor function | 11.54** | 1.24 | 9.10, 13.98 |  |  |
| *Item 29: Managing Money* | | | | | | | |
| A | Time | Managing Money | -0.43** | 0.06 | -0.55, 0.31 | 4.53** | 3.33** |
| B | Managing Money | Motor function | -7.71** | 1.16 | -9.98, -5.45 |  |  |
| C’ | Time | Motor function | 8.50** | 1.32 | 5.92, 11.09 |  |  |

*Significant to *p<*0.05; **Significant to *p<*0.001

**Table S5. Summary of the Level 1 Regression results testing the Mediational Effects of MPAI-4 Abilities Domain Items on Change in FIM+FAM Cognitive Score (n=211)**

| Path | Predictor Variable | Outcome Variable | B | SE B | 95% CI | Sobel Z | Path ab |
| --- | --- | --- | --- | --- | --- | --- | --- |
| C | Time | Cognitive function | 9.47** | 0.87 | 7.77, 11.16 |  |  |
| *Item 1: Mobility* | | | | | | |  |
| A | Time | Mobility | -0.44** | 0.06 | -0.56, -0.31 | 6.01** | 2.80** |
| B | Mobility | Cognitive function | -6.42** | 0.62 | -7.63, -5.21 |  |  |
| C’ | Time | Cognitive function | 6.67** | 0.87 | 4.97, 8.36 |  |  |
| *Item 2: Use of Hands* | | | | | | |  |
| A | Time | Use of hands | -0.40** | 0.06 | -0.52, -0.29 | 5.57** | 2.66** |
| B | Use of hands | Cognitive function | -6.60** | 0.65 | -7.87, -5.32 |  |  |
| C’ | Time | Cognitive function | 6.81** | 0.87 | 5.10, 8.51 |  |  |
| *Item 3: Vision* | | | | | | |  |
| A | Time | Vision | -0.31** | 0.64 | -0.43, -0.18 | 0.48 | 0.99* |
| B | Vision | Cognitive function | -3.20** | 0.69 | -4.56, -1.84 |  |  |
| C’ | Time | Cognitive function | 8.48** | 0.88 | 6.75, 10.21 |  |  |
| *Item 4: Audition* | | | | | | | |
| A | Time | Audition | -0.13* | 0.06 | -0.24, -0.02 | 1.85 | 0.41 |
| B | Audition | Cognitive function | -3.18** | 0.89 | -4.92, -1.43 |  |  |
| C’ | Time | Cognitive function | 9.05** | 0.87 | 7.35, 10.76 |  |  |
| *Item 5: Dizziness* | | | | | | | |
| A | Time | Dizziness | -0.26** | 0.06 | -0.38, -0.14 | - | - |
| B | Dizziness | Cognitive function | -1.06 | 0.83 | -2.68, 0.56 |  |  |
| C’ | Time | Cognitive function | 9.19** | 0.89 | 7.43, 10.94 |  |  |
| *Item 6: Motor Speech* | | | | | | | |
| A | Time | Motor Speech | -0.22** | 0.05 | -0.32, -0.12 | 3.96** | 1.23** |
| B | Motor Speech | Cognitive function | -5.66** | 0.62 | -6.87, -4.45 |  |  |
| C’ | Time | Cognitive function | 8.23** | 0.87 | 6.53, 9.93 |  |  |
| *Item 7: Verbal or Nonverbal Communication* | | | | | | | |
| A | Time | Verbal or Nonverbal Communication | -0.33** | 0.06 | -0.44, -.22 | 5.01** | 2.74** |
| B | Verbal or Nonverbal Communication | Cognitive function | -8.26** | 0.68 | -9.59, -6.92 |  |  |
| C’ | Time | Cognitive function | 6.73** | 0.87 | 5.02, 8.43 |  |  |
| *Item 8: Attention* | | | | | | | |
| A | Time | Attention | -0.29** | 0.07 | -0.44, -0.15 | 3.82** | 2.12** |
| B | Attention | Cognitive function | -7.20** | 0.73 | -8.63, -5.77 |  |  |
| C’ | Time | Cognitive function | 7.35** | 0.89 | 5.60, 9.10 |  |  |
| *Item 9: Memory* | | | | | | | |
| A | Time | Memory | -0.37** | 0.06 | -0.49, -0.26 | 5.47** | 3.16** |
| B | Memory | Cognitive function | -8.44** | 0.71 | -9.84, -7.04 |  |  |
| C’ | Time | Cognitive function | 6.31** | 0.86 | 4.62, 8.00 |  |  |
| *Item 10: Fund of Information* | | | | | | | |
| A | Time | Fund of Information | -0.45** | 0.08 | -0.60, -0.30 | 5.21** | 3.55** |
| B | Fund of Information | Cognitive function | -7.88** | 0.57 | -9.00, -6.76 |  |  |
| C’ | Time | Cognitive function | 5.92** | 0.88 | 4.19, 7.64 |  |  |
| *Item 11: Novel Problem Solving* | | | | | | | |
| A | Time | Novel problem-solving | -0.37** | 0.06 | -0.48, -0.26 | 5.51** | 3.68** |
| B | Novel problem-solving | Cognitive function | -9.95** | 0.81 | -11.53, -8.36 |  |  |
| C’ | Time | Cognitive function | 5.79** | 0.94 | 3.95, 7.62 |  |  |
| *Item 12: Visuospatial Abilities* | | | | | | | |
| A | Time | Visuospatial Abilities | -0.35** | 0.07 | -0.49, -0.21 | 4.32** | 1.81** |
| B | Visuospatial Abilities | Cognitive function | -5.25** | 0.61 | -6.44, -4.05 |  |  |
| C’ | Time | Cognitive function | 7.65** | 0.87 | 5.95, 9.34 |  |  |
| *Significant to *p<*0.05; **Significant to *p<*0.001 | | | | | | | |

**Table S6. Summary of the Level 1 Regression results testing the Mediational Effects of MPAI-4 Adjustment Domain Items on Change in FIM+FAM Cognitive Score (n=211)**

| Path | Predictor Variable | Outcome Variable | B | SE B | 95% CI | Sobel Z | Path ab |
| --- | --- | --- | --- | --- | --- | --- | --- |
| C | Time | Cognitive function | 9.47** | 0.87 | 7.77, 11.16 |  |  |
| *Item 13: Anxiety* | | | | | | |  |
| A | Time | Anxiety | -0.15 | 0.08 | -0.30, <0.001 | - | - |
| B | Anxiety | Cognitive function | -3.16** | 0.65 | -4.43, -1.90 |  |  |
| C’ | Time | Cognitive function | 8.99** | 0.86 | 7.30, 10.67 |  |  |
| *Item 14: Depression* | | | | | | |  |
| A | Time | Depression | -0.05 | 0.08 | -0.20, 0.10 | - | - |
| B | Depression | Cognitive function | -2.84** | 0.66 | -4.14, -1.54 |  |  |
| C’ | Time | Cognitive function | 9.33** | 0.85 | 7.66, 11.00 |  |  |
| *Item 15: Irritability, Anger, Aggression* | | | | | | |  |
| A | Time | Irritability, Anger, Aggression | 0.18* | 0.08 | 0.03, 0.33 | 2.13* | -0.77* |
| B | Irritability, Anger, Aggression | Cognitive function | -4.28** | 0.64 | -5.53, -3.03 |  |  |
| C’ | Time | Cognitive function | 10.24** | 0.85 | 8.58, 11.89 |  |  |
| *Item 16: Pain and Headache* | | | | | | | |
| A | Time | Pain and Headache | -0.22* | 0.07 | -0.36, -0.08 | 2.62* | 0.73* |
| B | Pain and Headache | Cognitive function | -3.29** | 0.69 | -4.63, -1.94 |  |  |
| C’ | Time | Cognitive function | 8.73** | 0.87 | 7.04, 10.43 |  |  |
| *Item 17: Fatigue* | | | | | | | |
| A | Time | Fatigue | -0.21* | 0.07 | -.035, -0.08 | 2.83* | 1.25* |
| B | Fatigue | Cognitive function | -5.85** | 0.69 | -7.21, -4.50 |  |  |
| C’ | Time | Cognitive function | 8.22** | 0.86 | 6.54, 8.90 |  |  |
| *Item 18: Sensitivity to Mild Symptoms* | | | | | | | |
| A | Time | Sensitivity to Mild Symptoms | -0.07 | 0.08 | -0.23, 0.10 | - | - |
| B | Sensitivity to Mild Symptoms | Cognitive function | -2.36** | 0.60 | -3.53, -1.18 |  |  |
| C’ | Time | Cognitive function | 9.31** | 0.84 | 7.66, 10.96 |  |  |
| *Item 19: Inappropriate Social Interaction* | | | | | | | |
| A | Time | Inappropriate Social Interaction | 0.10 | 0.08 | -0.07, 0.26 | - | - |
| B | Inappropriate Social Interaction | Cognitive function | -4.47** | 0.59 | -5.62, -3.32 |  |  |
| C’ | Time | Cognitive function | 9.89** | 0.82 | 8.28, 11.50 |  |  |
| *Item 20: Impaired Self-Awareness* | | | | | | | |
| A | Time | Impaired Self-Awareness | -0.16* | 0.08 | -0.32, -0.0006 | 1.92 | 0.65 |
| B | Impaired Self-Awareness | Cognitive function | -4.06** | 0.60 | -5.23, -2.88 |  |  |
| C’ | Time | Cognitive function | 8.81** | 0.83 | 7.18, 10.44 |  |  |
| *Item 21: Family/Significant Relationships* | | | | | | | |
| A | Time | Family/Significant Relationships | -0.17 | 0.09 | -0.35, 0.009 | - | - |
| B | Family/Significant Relationships | Cognitive function | -2.50** | 0.56 | -3.59, -1.41 |  |  |
| C’ | Time | Cognitive function | 9.04** | 0.86 | 7.36, 10.72 |  |  |
| *Significant to *p<*0.05; **Significant to *p<*0.001 | | | | | | | |

**Table S7. Summary of the Level 1 Regression results testing the Mediational Effects of MPAI-4 Participation Domain Items on Change in FIM+FAM Cognitive Score (n=211)**

| Path | Predictor Variable | Outcome Variable | B | SE B | 95% CI | Sobel Z | Path ab |
| --- | --- | --- | --- | --- | --- | --- | --- |
| C | Time | Cognitive function | 9.47** | 0.87 | 7.77, 11.16 |  |  |
| *Item 22: Initiation* | | | | | | |  |
| A | Time | Initiation | -0.45** | 0.08 | -0.61, -0.29 | 5.11** | 3.09** |
| B | Initiation | Cognitive function | -6.94** | 0.57 | -8.06, -5.81 |  |  |
| C’ | Time | Cognitive function | 6.37** | 0.86 | 4.69, 8.06 |  |  |
| *Item 23: Social Contact* | | | | | | |  |
| A | Time | Social Contact | -0.56** | 0.09 | -0.72, -0.39 | 4.87** | 2.64** |
| B | Social Contact | Cognitive function | -4.77** | 0.61 | -5.95, -3.58 |  |  |
| C’ | Time | Cognitive function | 6.82** | 0.91 | 5.03, 8.61 |  |  |
| *Item 24: Leisure and Recreation* | | | | | | |  |
| A | Time | Leisure and Recreation | -0.79** | 0.08 | -0.94, -0.64 | 6.37** | 4.66** |
| B | Leisure and Recreation | Cognitive function | -5.92** | 0.71 | -7.31, -4.54 |  |  |
| C’ | Time | Cognitive function | 4.80** | 1.02 | 2.81, 6.80 |  |  |
| *Item 25: Self-Care* | | | | | | | |
| A | Time | Self-Care | -0.53** | 0.08 | -0.68, -0.38 | 5.86** | 3.80** |
| B | Self-Care | Cognitive function | -7.15** | 0.57 | -8.26, -6.04 |  |  |
| C’ | Time | Cognitive function | 5.67** | 0.90 | 3.90, 7.44 |  |  |
| *Item 26: Residence* | | | | | | | |
| A | Time | Residence | -0.66** | 0.07 | -0.80, -0.53 | 7.04** | 5.26** |
| B | Residence | Cognitive function | -7.93** | 0.75 | -9.40, -6.46 |  |  |
| C’ | Time | Cognitive function | 4.20** | 1.00 | 2.25, 6.16 |  |  |
| *Item 27: Transportation* | | | | | | | |
| A | Time | Transportation | -0.60** | 0.06 | -0.73, -0.48 | 6.91** | 4.26** |
| B | Transportation | Cognitive function | -7.08** | 0.74 | -8.53, -5.63 |  |  |
| C’ | Time | Cognitive function | 5.20** | 0.96 | 3.33, 7.08 |  |  |
| *Item 28: Paid or Other Employment* | | | | | | | |
| A | Time | Paid or Other Employment | -0.06 | 0.04 | -0.14, 0.03 | - | - |
| B | Paid or Other Employment | Cognitive function | -5.30** | 1.10 | -7.45, -3.14 |  |  |
| C’ | Time | Cognitive function | 9.16** | 0.85 | 7.50, 10.83 |  |  |
| *Item 29: Managing Money* | | | | | | | |
| A | Time | Managing Money | -0.43** | 0.06 | -0.55, -0.31 | 5.76** | 3.22** |
| B | Managing Money | Cognitive function | -7.46** | 0.77 | -8.96, -5.96 |  |  |
| C’ | Time | Cognitive function | 6.25** | 0.91 | 4.46, 8.04 |  |  |

*Significant to *p<*0.05; **Significant to *p<*0.001
